# The burden of acute respiratory infection in children under 5 attributable to economic inequality in low- and middle-income countries

**DOI:** 10.1136/bmjgh-2024-017409

**Published:** 2025-03-07

**Authors:** Hailu Zhu, Ke Huang, Xueyan Han, Zhaoyang Pan, Hanchao Cheng, Qi Wang, Yicong Wang, Wei Sun, Jiarun Mi, Ting Yang, Tianjia Guan, Tao Xue, Chen Wang

**Affiliations:** 1 School of Health Policy and Management, Chinese Academy of Medical Sciences and Peking Union Medical College, Beijing, China; 2 National Center for Respiratory Diseases; State Key Laboratory of Respiratory Health and Multimorbidity; National Clinical Research Center for Respiratory Diseases; Institute of Respiratory Medicine, Chinese Academy of Medical Sciences; Department of Pulmonary and Critical Care Medicine, Center of Respiratory Medicine, China-Japan Friendship Hospital, Beijing, China; 3 Institute of Reproductive and Child Health / National Health Commission Key Laboratory of Reproductive Health and Department of Epidemiology and Biostatistics / Ministry of Education Key Laboratory of Epidemiology of Major Diseases (PKU), School of Public Health, Peking University Health Science Centre, Beijing, China; 4 Advanced Institute of Information Technology, Peking University, Hangzhou, China; 5 State Environmental Protection Key Laboratory of Atmospheric Exposure and Health Risk Management, Center for Environment and Health, Peking University, Beijing, China; 6 Chinese Academy of Medical Sciences and Peking Union Medical College, Beijing, China

**Keywords:** Epidemiology, Child health, Cross-sectional survey, Global Health

## Abstract

**Background:**

Quantifying the disease burden among children that could potentially be reduced through improvements in individual economic status and regional economic equality can greatly benefit policy making and resource allocation. However, such quantification has rarely been done. This study aimed to assess the inequality-related burden of acute respiratory infection (ARI) (the leading cause of child mortality in low- and middle-income countries (LMICs)) among under five children.

**Methods:**

This study integrated the Demographic and Health Survey data from 53 countries and linked individual records to a novel proxy of economic development status, the satellite night-time light (NTL). We assessed the number of children affected by ARI attributable to within-country economic disparities (eg, NTL<the country-specific 90th percentile) or within-country inequality (eg, NTL<the annual country-specific 90th percentile) from 2001 to 2019 in 133 LMICs, based on the exposure-response relationship between NTL and ARI derived from the study participants.

**Results:**

The odds of experiencing ARI were decreased significantly (3.5% ((95% confidence interval (CI) 1.4% to 4.4%)) for every 10-digit number increase in NTL. The exposure–response function showed constant decreasing in the risk of ARI as NTL level increases. It is estimated that within-country economic disparities contributed to 11.0% (95% CI 6.1% to 15.6%) of all children affected by ARI in 2001, which was decreased to 8.1% (95% CI 4.2% to 11.8%) in 2019. In contrast, the inequality-related burden remained stable. In sub-Saharan Africa, it increased from 4.8% (95% CI 1.7% to 8.0%) in 2001 to 6.8% (95% CI 3.0%−9.8%) in 2019. Eliminating within-country inequality would have avoided 522 136 (95% CI 2 66 760 to 7 57 414) cases of ARI among children across the 133 LMICs in 2019.

**Conclusion:**

Our study revealed a protective effect of economic status on preventing ARI in children under 5 years of age. The considerable burden of childhood ARI was attributable to the economic inequality in LMICs. Optimising the allocation of economic resources can safeguard child health.

**WHAT IS ALREADY KNOWN ON THIS TOPIC**

- The WHO has emphasised that economic inequality, particularly its impact on children’s health, is likely one of the ‘true determinants’ of health inequality. However, the actual impact of economic inequality on disease burden has rarely been examined. Acute respiratory infections (ARI)—one of the primary causes of child mortality—exhibit significant variations in prevalence and improvement across different countries, particularly in low- and middle-income countries (LMICs). Therefore, it could be of importance to investigate the impact of economic inequality on ARI in children.

**WHAT THIS STUDY ADDS**

- Using ARI data from the Demographic Health Survey and satellite night-time light (NTL) intensity as a novel proxy of economic development, we found a negative association between the risk of ARI and economic status in LMICs. We also discovered that the ARI burden attributable to within-country inequality (eg, NTL<the annual country-specific 90th percentile) consistently increased from 2001 to 2019, especially in sub-Saharan Africa.

**HOW THIS STUDY MIGHT AFFECT RESEARCH, PRACTICE OR POLICY**

- This study revealed a protective effect of economic status on preventing ARIs in children under five years of age. The considerable burden of childhood ARIs was attributable to the economic inequality in LMICs. Optimising the allocation of economic resources may safeguard child health.

## Introduction

 Over the past two decades, with economic development, significant progress has been made in improving child health. Despite this encouraging advancement, progress remains uneven both among and within countries, particularly in low- and middle-income nations (LMICs).^1^ Several studies have highlighted that economic inequality may be a key factor contributing to health disparities.^1^,^3^ As reported by the WHO, the likelihood of a child dying under the age of five is 14 times higher in sub-Saharan Africa than in other countries worldwide.^4^ Health inequalities attributed to economic inequality not only occur between countries but are also likely to occur within countries and even within specific regions. In India, the absolute gap in under-5 mortality rates between the poorest and wealthiest households has significantly decreased since 1990, while the relative differences have stayed constant.^5^ Therefore, when considering the economic impact on health, it is important to focus on the effects of both economic disparities and relative inequalities.

Acute respiratory infection (ARI) represents a significant cause of mortality in children under five worldwide. Approximately 15% of global under-5 mortality is attributed to ARIs, the majority of which occur in LMICs.^6^ Although ARI is the leading cause of child mortality in LMICs, the association between economic inequality and ARI has not been studied. In addition, the lack of reliable household income data in many areas makes it difficult to quantify economic inequality.^7 8^ Traditional methods of measuring inequality also have limitations in that they cannot be effectively compared across different geographical scales,^9 10^ which limit their applicability in subregional studies. Thus, to accurately identify the economic inequality, we employ night-time light (NTL) as a quantitative indicator. NTL has been shown to be closely linked to human economic activity and to reflect economic inequality,^11^ making it a suitable representative of economic levels.^12^,^14^

This study used multicentre, cross-national data to assess the association between economic status and ARI in children under five. Furthermore, using exposure–response function, we estimated the disease burden attributed to economic disparities or relative economic inequality among children under five in 133 LMICs.

## Methods

### Demographic data

Demographic data were collected from the Demographic and Health Surveys (DHS). This programme used a two-stage stratified cluster sampling design to collect nationally representative household sample data from several developing countries. In the first stage, proportional probability sampling was used to select survey areas for stratified sampling. In the second stage, within the selected survey areas, households were selected with equal probability. A comprehensive survey was then conducted for each selected household. The investigators also collected relevant information on the health and medical history of their offspring born within the 5 years prior to the survey date by interviewing the female members of the households aged 15 to 49 years. In addition, the programme used the Global Positioning System (GPS) to collect geographic location information on the sampled population. The official website and previous publications have published the details of the database.^15^,^17^ Children were excluded from the study due to death, missing exposure or outcome data or insufficient information on sample stratification. The study ultimately included 1 281 847 children in 53 LMICs from 2001 to 2019 (online supplemental table S1).

### Environmental data

In this study, we used NTL data as a proxy for economic development. The NTL data are derived from a worldwide database of NTL time series.^18^ This database was compiled by integrating calibrated NTL observations from the Defense Meteorological Satellite Program (DMSP) data with simulated DMSP-like NTL observations from the Visible Infrared Imaging Radiometer Suite (VIIRS) data, aiming to produce a comprehensive and consistent NTL dataset globally. To obtain a consistent NTL time series database, the kernel density matching method for NTL data transforms VIIRS NTL remote sensing data to resemble DMSP annual NTL remote sensing data. Then, it is integrated with DMSP NTL remote sensing data obtained through a stepwise calibration approach to produce the new annual NTL remote sensing dataset. The primary resolution of the NTL data is 1 km × 1 km. To safeguard the personal privacy of the respondents, the DHS survey randomly shifted GPS coordinates, leading to positional errors ranging from 0 to 10 kilometres.^19^ To match with the addresses, we aggregated the NTL data within each 10 km × 10 km grid. We matched the corresponding NTL based on the geographic information of each participant. To minimise the possibility of exposure misclassification, we used NTL in the year of the survey as the primary exposure time window (Lag1).

### Outcome variables and covariates

The outcome variable, ARI, was defined as ‘short, rapid breathing that was chest-related and/or difficult breathing that was chest-related’. The definition of ARI in the DHS focuses primarily on chest symptoms and is more likely to reflect individuals with acute lower respiratory infection. According to the official guide for DHS data, a child under 5 was considered to have had ARI if their mother reported that the child had experienced these symptoms in the 2 weeks before the survey. We also included other risk factors in the analysis, including characteristics related to demographic characters, pregnancy, reproductive, maternal and household characteristics (online supplemental table S1, text1).

### Statistical analyses

To evaluate the association between economic status and ARI in children under five, this study employed a linear fixed-effects model for analysis. For continuous variables such as household head age, maternal age and pregnancy interval, we used cubic spline functions to evaluate their potential nonlinear effects. In cases where there were missing values among the covariates, this study estimated them using a chained equation approach. According to the official protocol for DHS data preparation, we weighted participants using country-specific household survey weights (obtained from the DHS) to ensure the representativeness of the estimates and adjust for selection bias. We used the OR, expressed as OR=exp(10β), to assess the ratio of the increase in respiratory infection rates for every 10-digit increment in NTL (DN) values. To assess the robustness of the model, sensitivity analyses were conducted by using different lag periods and adjusting medical treatment or advice. We further applied a method that we had previously published for multicentre epidemiological analysis to develop a nonlinear exposure‒response function (online supplemental text2). This was achieved by integrating the estimated marginal effects of NTL from different strata.^20^

### The risk assessment of economic disparities and economic inequality

Based on previous studies,^21 22^ when assessing the burden of disease attributed to within-country economic disparities, we used the population-weighted 90th percentile of NTL values for each country in 2019 as the reference. To assess the burden of disease attributed to relative economic inequality, we used the population-weighted 90th percentile of annual NTL levels for global, individual countries or regions as a reference. Considering the varying distribution of geographic resources in each country, the results primarily focused on the burden of disease attributed to within-country economic disparities and relative economic inequalities.

Based on the exposure‒response association estimated in this study, we conducted a risk assessment in a 0.1°×0.1° grid, covering 133 LIMCs from 2001 to 2019. When performing point estimates, the use of different reference values did not change the curvature of the exposure–response function, but only caused a vertical shift in the graph. Therefore, in this study, it was only necessary to conduct the appropriate numerical conversions for calculating attributable fractions using different reference values. We employed the following equation to calculate the attributable fraction of ARI under five for each area:

$$AF_{g,t}=1-1/(exp\;[f(x_{g,t})]/\;exp\;[f(x_{g^{c},t})]),\;AN_{g,t}=AF_{g,t}\times N_{g,t}$$

where g represents the spatial grid index, t denotes the calendar year index, $x_{g,t}$ is the level of NTL, $x_{g^{c},t}$ is the 90th percentile level of NTL, $f\left(x_{g,t}\right)$ represents the corresponding OR obtained based on the exposure–response function, $N_{g,t}$ is the baseline count of ARI in children under 5, and ${AF}_{g,t}$ and ${AN}_{g,t}$ represent the attributable fraction and the attributable number respectively. We aggregated data on the prevalence of ARI in children under five from 133 LMICs. The data spanned from 2001 to 2019 and were obtained from the Global Burden of Disease Study 2019 Results. Therefore, based on previous research,^23^,^27^ this study approximated the number of ARIs attributed to economic disparities or relative economic inequality among children under five in 133 low- and middle-income countries from 2001 to 2019. All the statistical analyses were conducted in R (V.4.3.1).

## Results

**Figure 1:**
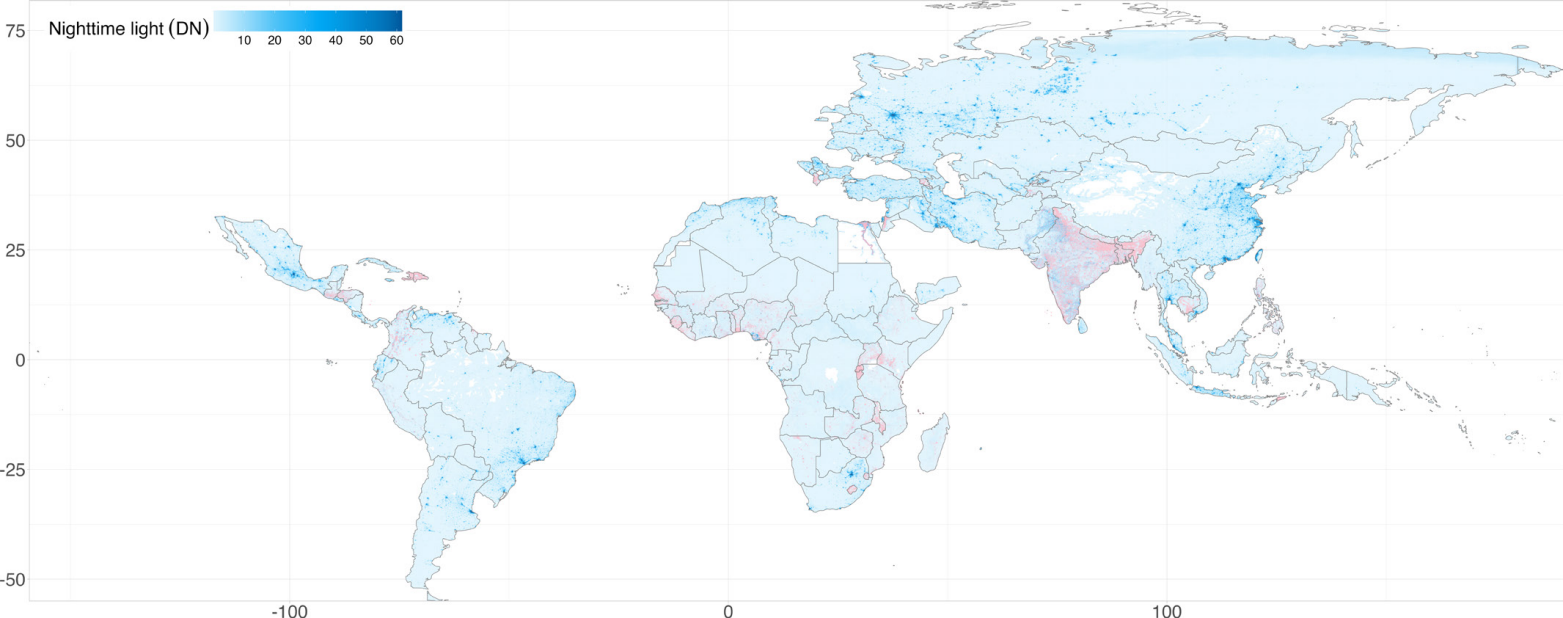
Study area for risk assessment (grey polygons), geographical locations of samples analysed (pink dots) and long-term average night-time light levels in human settlements (background colours).

The study included 1 281 847 children under five from 53 countries, among whom 60 610 were reported to have experienced an ARI within 2 weeks prior to the survey date. The areas where children with ARI resided exhibited lower levels of NTL than did the unaffected group. The average age of the children was 2 years and 4 months, with 50.9% being boys. Additionally, more than half of the children (51.8%) were born in hospitals. Male heads of household predominated (82.7%) with an average age of 42.3 (SD=14.2) years, while the average age of the children’s mothers was 26.5 (SD=6.3) years. Figure 1 shows the distribution of NTL exposure across all areas. Detailed information on the study population can be found in online supplemental Table S2.

To examine the strength of the associations between economic status and the risk of ARI in children under 5, a linear fixed-effects analysis was performed (online supplemental figure S1). After adjusting the weights to control for selection bias and fixed effects in the model, each 10-digit number (DN) increase in NTL was negatively associated with ARI (OR=0.96, 95% CI 0.95 to 0.98). After further adjustment for demographic, pregnancy, reproductive, maternal and household characteristics, this association still supported this association: every 10DN in NTL was significantly associated with a lower risk of ARI in the fully adjusted model (OR=0.97, 95% CI 0.95 to 0.98). We used different lag periods to assess the robustness of the associations (online supplemental figure S2). The results showed that the association did not change significantly. Additionally, the results of the stratified effect modification analysis indicate that household factors did not significantly alter the association between economic status and ARI in children under 5. Additional results of sensitive and subgroup analyses are presented in the online supplemental figure S3–S4.

**Figure 2:**
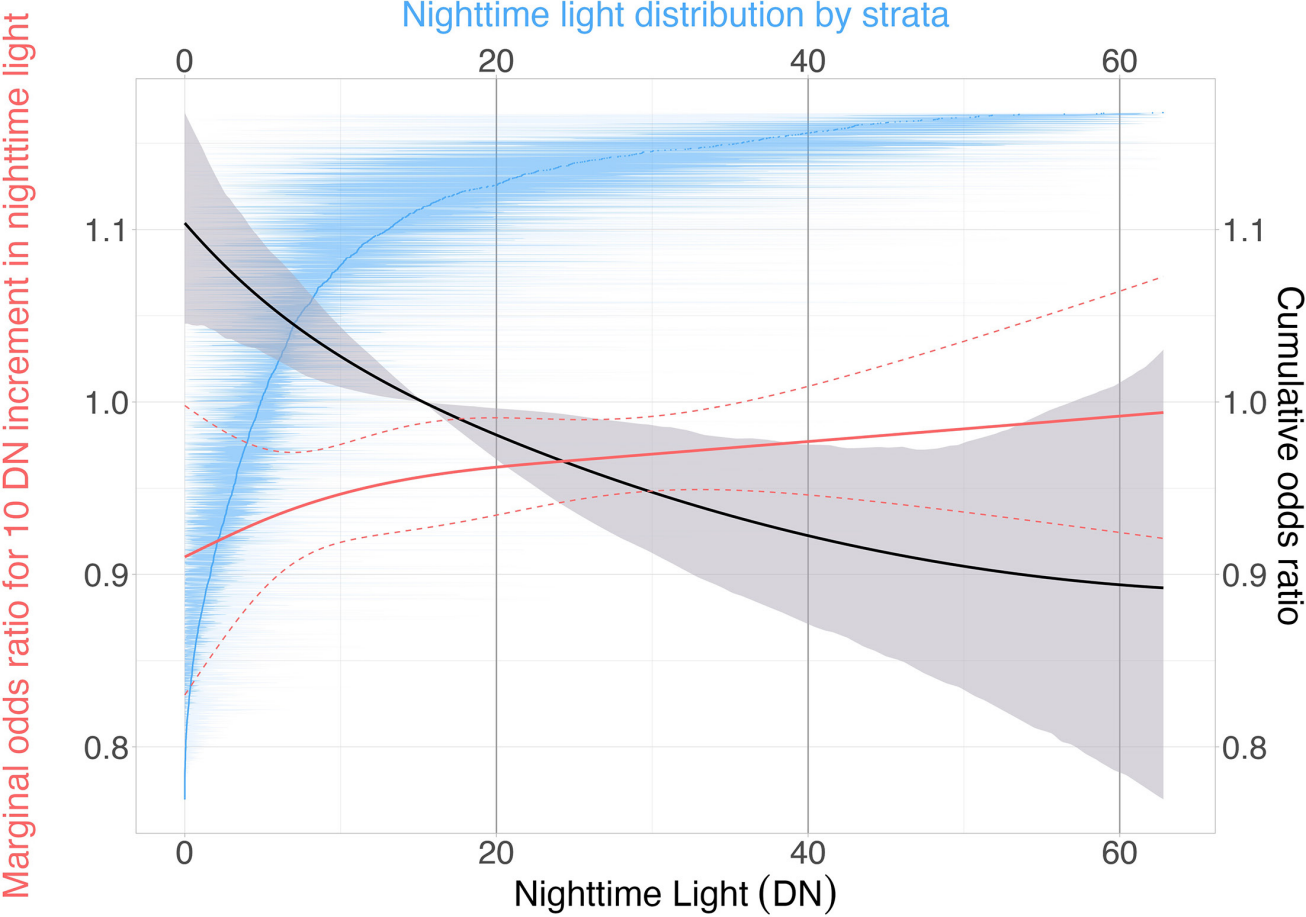
Non-linear exposure–response function for the economy and acute respiratory infection in children under five. The black line is the estimated exposure‒response function. The red lines represent the first derivatives of the function (solid line) and their 95% CIs (dashed lines). The blue line shows the cumulative population distribution by stratum-level average nighttime light level, and its corresponding ribbons show the 95% CI (blue).

By integrating the marginal effects of NTL estimated from different strata, we established a nonlinear relationship between economic factors and ARI in children under 5, using a fully adjusted model. The non-linear results suggest that with increasing economic status, the risk of ARI in children under five gradually decreases, which is consistent with the findings of the linear fixed-effects model (figure 2). Additionally, the risk of ARI among children under five decreases with increasing economic status.

**Figure 3:**
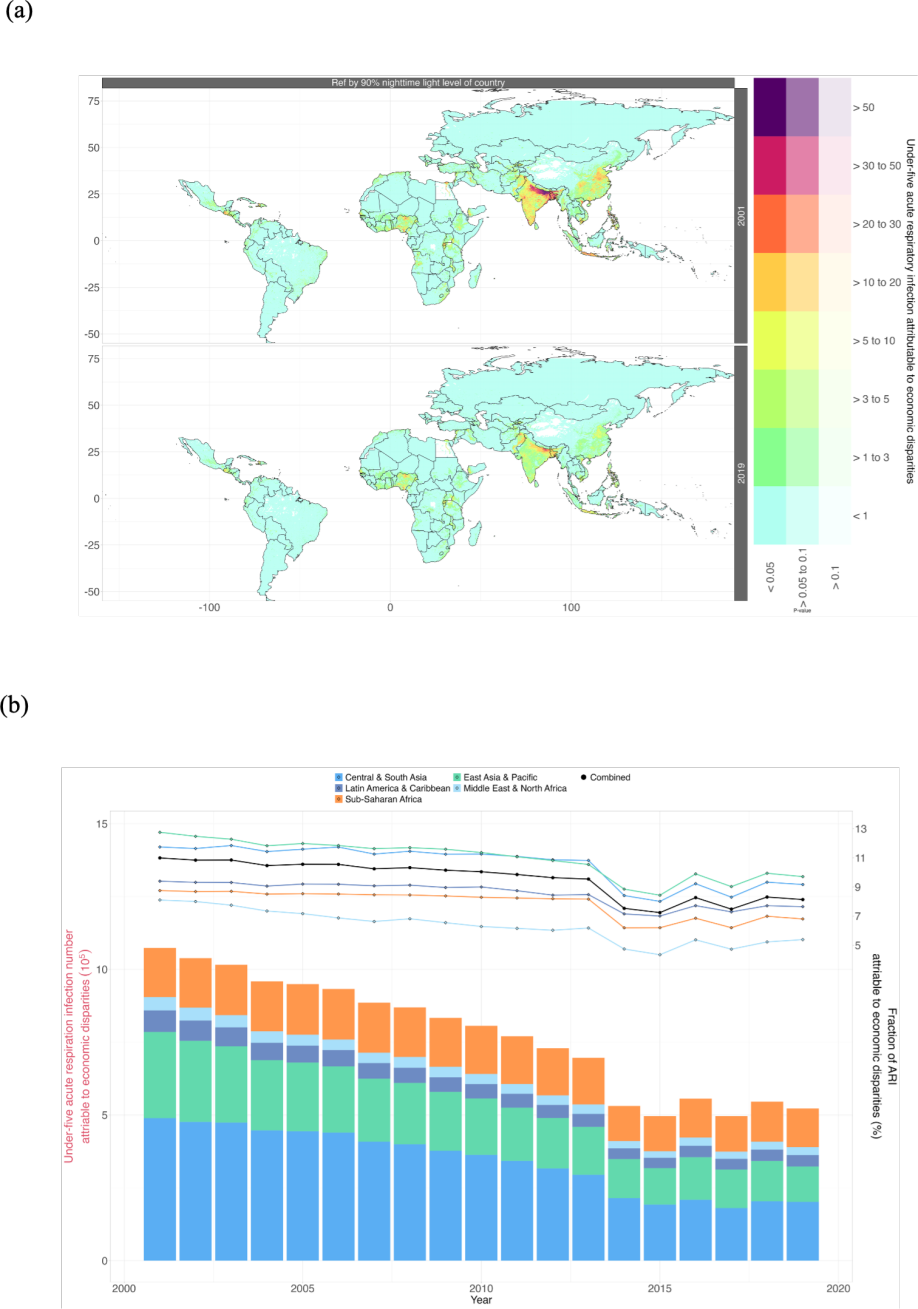
(**a**) The acute respiratory infection burden in children under five attributed to within-country economic disparities in 133 low- and middle-income countries (LMICs) in 2001 and 2019. (**b**) The temporal trends of acute respiratory infection burden in children under five are attributable to within-country economic disparities, segmented by geographic location. The left y-axis represents the attributable numbers (coloured bars), and the right y-axis represents the attributable fractions (circles and lines). ARI, acute respiratory infection.

Based on the exposure‒response function, we initially evaluated the number of ARI among children under five attributable to within-country economic disparities within 133 LMICs during the period from 2001 to 2019 (figure 3(a)). Within-country economic disparities in 133 LMICs contributed to a total of 1 073 660 cases (95% CI 600 010 to 1 521 070), accounting for 11.0% (95% CI 6.1% to 15.6%) of all children affected by ARI in 2001. As a result of economic growth, the number or proportion of ARI attributable to within-country economic disparities in 133 LMICs declined to 522 136 (95% CI 266 760 to 757 414) or 8.1% (95% CI 4.2% to 11.8%) in 2019 (figure 3b, online supplemental figure S5). In India, the number of children under five with ARI attributed to within-country economic disparities experienced the most substantial decline, dropping from 370 989 (95% CI 192 603 to 528 382) in 2001 to 124 509 (95% CI 46 659 to 181 994) in 2019. In addition, in China, the number decreased from 187 135 (95%CI: 74 375 to 3 00 051) in 2001 to 68,161 (95%CI: 18 529 to 1 17 598) in 2019 (online supplemental figure S5). The regional distribution of the burden of ARI attributed to within-country economic disparities from 2001 to 2019 is depicted in figure 3b. During this period, all regions showed an overall decreasing trend in the burden attributable to within-country economic disparities, with the most significant declines observed in East Asia and the Pacific, as well as Central and South Asia. The burden of acute lower respiratory infection in terms of within-world economic disparities and within-region economic disparities also exhibited similar trends, as detailed in online supplemental figure S7–S8.

**Figure 4:**
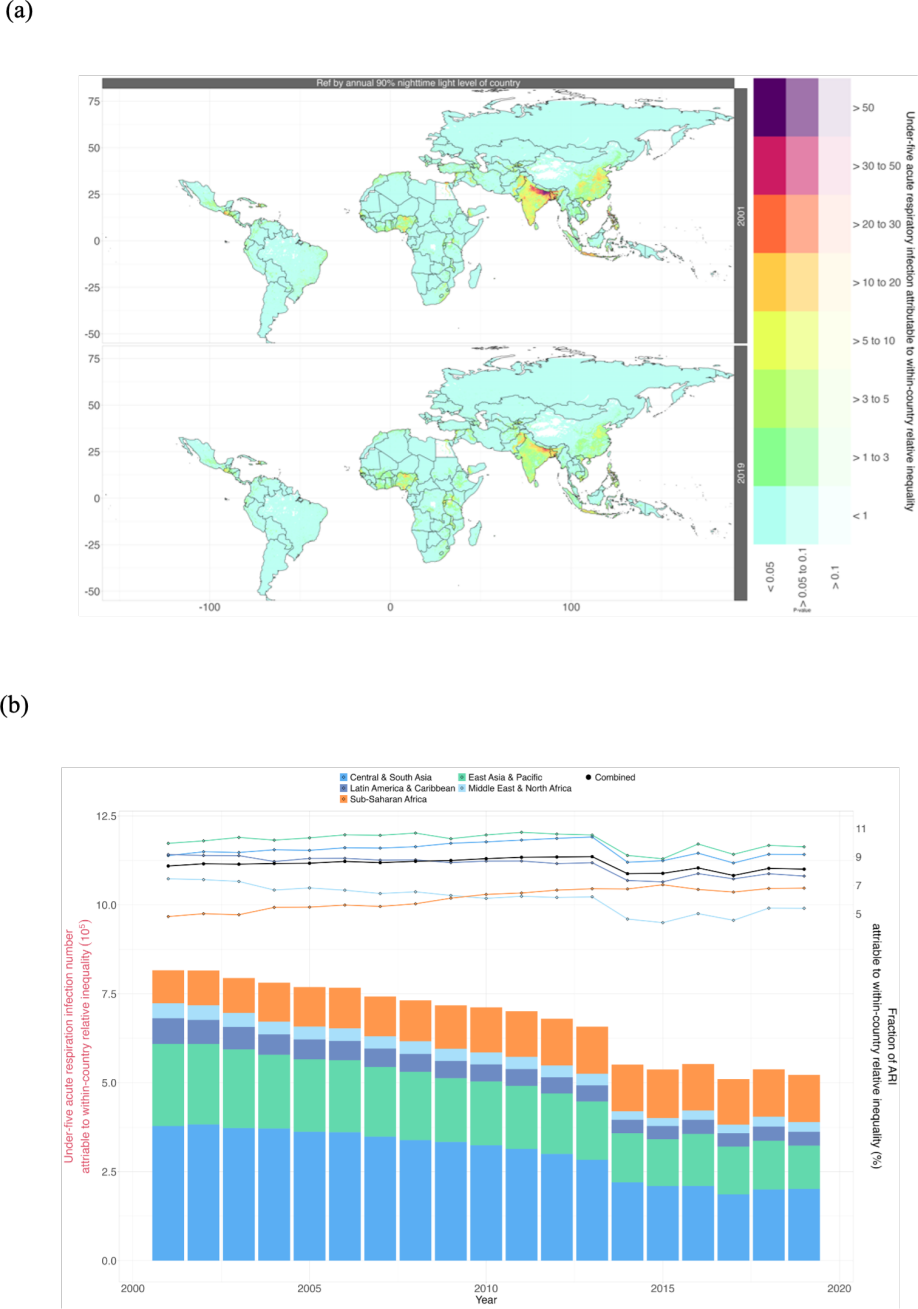
(**a**) The burden of acute respiratory infection among children under 5 attributed to within-country relative economic inequality in 133 low- and middle-income countries in 2001 and 2019. (**b**) The temporal trends of acute respiratory infection burden in children under 5, attributed to within-country relative economic inequality, are segmented by geographic location. The left y-axis represents the attributable numbers (coloured bars), and the right y-axis represents the attributable fractions (circles and lines). ARI, acute respiratory infection.

The spatial distribution of the burden of ARI in 133 LMICs attributed to within-country relative economic inequality from 2001 to 2019 is depicted in figure 4a. While the number of ARIs among children under 5 attributed to within-country relative economic inequality has declined, the attributable fraction has shown an increasing trend. This trend was particularly pronounced in Sub-Saharan Africa, where the attributable fraction increased from 4.8% (95% CI 1.7% to 8.0%) in 2001 to 6.8% (95% CI 3.0% to 9.8%) in 2019 (figure 4b). Specifically in Tanzania, the fraction attributable to ARI increased significantly from 3.1% (95% CI 1.0% to 5.5%) in 2001 to 7.0% (95% CI 3.8% to 10.4%) in 2019. Additionally, in Kenya and Uganda, the attributable fraction also showed a significant increase. Notably, in Indonesia, the attributable fraction remained consistently high from 2001 (10.6%, 95% CI 5.4% to 15.1%) to 2019 (10.0%, 95% CI 3.6% to 16.5%). Similar trends were also found in China (from 10.3% (95% CI 5.7% to 15.1%) in 2001 to 10.1% (95% CI 3.0% to 17.3%) in 2019) and India (from 9.6% (95% CI 5.5% to 14.0%) in 2001 to 9.4% (95% CI 3.7% to 14.4%) in 2019). The burden of ARI related to within-world relative inequality and within-region relative inequality exhibited similar trends (see online supplemental figure S6, S9–S11).

## Discussion

In this multicentre epidemiological study, we focused on exploring the impact of the economy on ARI in children under 5 in LMICs. We further evaluated the burden of ARI on children attributable to economic disparities or relative economic inequality in 133 LMICs. Our research suggested an association between higher economic status and a lower risk of ARI in children under 5 (OR=0.97, 95% CI 0.95 to 0.98). It is worth noting that, although there has been a decrease in the number of cases of ARI in children under 5 attributed to economic disparities or relative economic inequality, the attributable fraction associated with relative economic inequality has shown an increasing trend.

Previous studies have demonstrated an association between economic status and children’s respiratory health. Yaya *et al*, by categorising household wealth into five groups, found that children under 5 from the wealthiest families had a significantly lower risk of ARI than did those from the least wealthy families.^28^ Additionally, Ekholuenetale *et al*, using data from 37 countries, also found a significant negative association between the household wealth index and the risk of ARI in children under 5.^29^ In our study, we further demonstrated through an exposure–response function that the risk of ARI in children under 5 years of age tends to decrease as the economic level increases. This may be because higher economic levels tend to be associated with improved hygiene conditions and practices. In addition, areas with a higher economic status often offer better educational opportunities, which promote greater awareness and adherence to preventive health measures within families, thereby reducing the prevalence of respiratory infection in children.^30^

With economic development, for both global, national and regional comparisons, the number of children under 5 suffering from ARI attributed to economic disparities in 133 LMICs has shown a marked downward trend, accompanied by a corresponding decrease in the attributable fractions. In Central and South Asia, the primary reduction was predominantly driven by outcomes from India, given that its population size is the largest among all countries in the region. Similarly, in East Asia and the Pacific region, the primary reduction in disease burden was mainly driven by outcomes from China. In addition to the large populations of these two countries, their rapid economic development is also a major factor.^31^ A higher level of economic development has further facilitated the improvements in basic healthcare and services in these countries, thereby reducing the number of children suffering from ARI. Moreover, the decline in the attributable fraction also confirms a decline in the level of economic disparities. Mirza MU *et al* also found the same trend using NTL data to measure economic inequality.^11^ This phenomenon may reflect a more equitable distribution of social resources and improvements in the health system, resulting in an overall reduction in the impact of economic disparities on the number of children suffering from ARI.

During the period from 2001 to 2019 in 133 LMICs, it is notable that the attributable fraction of relative economic inequality was stable or even increasing, both in regions with rapid economic development and in those with slower growth rates. At the regional level, Sub-Saharan Africa experienced a significant increase in the number of children under 5 with ARI attributed to economic relative inequality, along with a corresponding increase in attributable fractions. Specifically, in Pakistan, the number of children under 5 with ARI significantly increased due to relative economic inequality, along with a substantial increase in the corresponding attributable fraction. Furthermore, in Kenya and Indonesia, the attributable fractions related to relative economic inequality exhibited an upward trend. Although in India and China, the number of children under 5 with ARI attributed to relative economic inequality significantly decreased, and the attributable fractions remained nearly unchanged. With economic development post-2019, the degree of relative economic inequality may further increase, highlighting the need for future policy interventions to mitigate the widening health impacts caused by the continuous expansion of relative economic inequality, especially in Sub-Saharan Africa.

This study has several limitations. First, our analysis was based on a cross-sectional study design, which provides limited evidence for establishing causal relationships. This type of research design may have some general limitations. Despite adjusting for multiple covariates and covering various aspects, there may still be some potential unmeasured confounding factors. In addition, since the specific cause of death data were not available in the DHS database, we did all our estimations among living children under 5; this could bring survival bias. Second, we used NTL data to measure economic income, but it is important to note that light data can be influenced by other factors, such as energy-saving policies, potentially leading to an underestimation of economic inequality. However, this challenge is not unique to light data; self-reported income faces similar dilemmas.^10^ For example, wealthier individuals may be inclined to underreport their income, making it difficult for survey-based assessments to accurately capture the highest income levels and leading to an underestimation of true inequality. Furthermore, although using NTL as a proxy of economic status has many advantages, especially in the between-country comparisons, countries with little variation in urban development should be cautious in using this indicator to assess within-country economic inequality. Despite these challenges, existing research suggests a significant correlation between economic inequality measured using night light data and income-based measures of economic inequality.^11^ Third, the outcome variable was based on reports from mothers of children; thus, the data may have been affected by recall or information bias. According to the official guidelines for DHS data, there is a significant amount of missing data in the outcome, which is recommended to be classified as controls. This could lead to misclassification of outcomes, potentially resulting in underestimation of the association. To assess the burden of disease, we used the prevalence of ARI rather than the incidence, which may have also introduced a prevalence-incidence bias. However, as the prevalence in this study was less than 5%, epidemiological theory suggests that these two measures may be approximately interchangeable.^32^ Finally, our assessment was limited to 133 LMICs from 2001 to 2019. And the validity of our exposure–response function may be limited for the disease burden assessment, because our estimates of the disease burden relied on the assumption that the exposure–response function derived from 53 DHS countries is universal and applies to 133 LMICs. Besides, the population data from GBD 2019 used to calculate the attributable number has certain limitations, such as reliance on model-based estimates for countries with insufficient registration data, a factor that has been extensively discussed in previous literature.^33 34^ Therefore, the use of GBD data could introduce bias into our study when calculating the attributable number. Caution should be exercised when interpreting the representativeness of our conclusions globally and across different time periods.

Using data from 53 LMICs, our study results revealed a protective effect of economic development on preventing ARI in children under 5 years of age. We also showed that a considerable burden of childhood ARI was attributed to the economic disparities and relative inequality in 133 LMICs. In addition to considering the impact of economic disparities on ARI, policymakers should pay particular attention to the influence of relative economic inequality. At the regional level, emphasis should be placed on the impact of the relative economic inequality on the health of children under 5 in Sub-Saharan Africa.

## supplementary material

10.1136/bmjgh-2024-017409 online supplemental file 1

## Data Availability

Data are available upon reasonable request.
