# Supplementary material for: The burden of acute respiratory infection in children under 5 attributable to economic inequality in low- and middle-income countries
Source: BMJ Glob Health. 2025 Mar 7;10(3):e017409. doi: 10.1136/bmjgh-2024-017409 (PMC11891534; doi:10.1136/bmjgh-2024-017409)
Supplement: online supplemental file 1 [file bmjgh-10-3-s001.pdf]

The burden of acute respiratory infection in children under five attributable to economic inequality in low- and middle-income countries

Hailu Zhu[1†], MS; Ke Huang[2†], MD; Xueyan Han[1], MPH; Zhaoyang Pan[1], BM; Hanchao Cheng[1], MS; Qi Wang[1], BM; Yicong Wang[1], BM; Wei Sun[1], BM; Jiarun Mi[1], BM; Ting Yang[2], PhD; Tianjia Guan[1*], PhD; Tao Xue[3,4,5*], PhD; Chen Wang, MD[2,6]

1. School of Health Policy and Management, Chinese Academy of Medical Sciences and Peking Union Medical College, Beijing, China;
2. National Center for Respiratory Diseases; State Key Laboratory of Respiratory Health and Multimorbidity; National Clinical Research Center for Respiratory Diseases; Institute of Respiratory Medicine, Chinese Academy of Medical Sciences; Department of Pulmonary and Critical Care Medicine, Center of Respiratory Medicine, China-Japan Friendship Hospital, Beijing, P. R. China.
3. Institute of Reproductive and Child Health / National Health Commission Key Laboratory of Reproductive Health and Department of Epidemiology and Biostatistics / Ministry of Education Key Laboratory of Epidemiology of Major Diseases (PKU), School of Public Health, Peking University Health Science Centre, Beijing, China
4. Advanced Institute of Information Technology, Peking University, Hangzhou, China;
5. College of Environmental Sciences and Engineering, Peking University Beijing, China. Peking University;
6. Chinese Academy of Medical Sciences and Peking Union Medical College, Beijing, P. R. China.

[†]These authors contributed equally to this work.

*Corresponding to Tianjia Guan (gtj@sph.pumc.edu.cn) and Tao Xue (txue@hsc.pku.edu.cn).

Table S1 Surveys and their sample sizes included in the present study.

DHS country code	survey	Sample size
AL	Albania Standard DHS, 2008	1586
	Albania Standard DHS, 2017	2755
AO	Angola Standard DHS, 2015	13619
AM	Armenia Standard DHS, 2010	1450
	Armenia Standard DHS, 2015	1710
BD	Bangladesh Standard DHS, 2007	5789
	Bangladesh Standard DHS, 2011	8332
	Bangladesh Standard DHS, 2014	7567
	Bangladesh Standard DHS, 2017	8402
BJ	Benin Standard DHS, 2011	12679
	Benin Standard DHS, 2017	12651
BF	Burkina Faso Standard DHS, 2010	13716
BU	Burundi MIS, 2010	7231
	Burundi Standard DHS, 2016	12472
KH	Cambodia Standard DHS, 2005	7695
	Cambodia Standard DHS, 2010	7820
	Cambodia Standard DHS, 2014	6971
CM	Cameroon Standard DHS, 2011	10734
	Cameroon Standard DHS, 2018	9085
TD	Chad Standard DHS, 2014	16901
CO	Colombia Standard DHS, 2010	17443
KM	Comoros Standard DHS, 2012	3022
CD	Congo Democratic Republic Standard DHS, 2013	17228
CI	Cote d'Ivoire Standard DHS, 2011	7093
DR	Dominican Republic Special DHS, 2013	866
	Dominican Republic Standard DHS, 2007	10796
	Dominican Republic Standard DHS, 2013	3606
EG	Egypt Standard DHS, 2008	10595

	Egypt Standard DHS, 2014	15466
SZ	Eswatini Standard DHS, 2006	2537
ET	Ethiopia Standard DHS, 2011	10808
	Ethiopia Standard DHS, 2016	10006
GA	Gabon Standard DHS, 2012	5747
GH	Ghana Standard DHS, 2008	2794
	Ghana Standard DHS, 2014	5595
GU	Guatemala Standard DHS, 2014	12071
GN	Guinea Standard DHS, 2012	6424
	Guinea Standard DHS, 2018	7273
GY	Guyana Standard DHS, 2009	2105
HT	Haiti Standard DHS, 2005	5596
	Haiti Standard DHS, 2012	6744
	Haiti Standard DHS, 2016	6120
HN	Honduras Standard DHS, 2011	10592
IA	India Standard DHS, 2015	247735
	India Standard DHS, 2019	102704
JO	Jordan Standard DHS, 2007	10237
	Jordan Standard DHS, 2012	10128
	Jordan Standard DHS, 2017	10475
KE	Kenya Standard DHS, 2008	5706
	Kenya Standard DHS, 2014	20093
KY	Kyrgyz Republic Standard DHS, 2012	4247
LS	Lesotho Standard DHS, 2009	3606
	Lesotho Standard DHS, 2014	2915
LB	Liberia Standard DHS, 2007	5305
	Liberia Standard DHS, 2013	7058
	Liberia Standard DHS, 2019	2988
MD	Madagascar Standard DHS, 2008	11750
MW	Malawi Standard DHS, 2010	18360
	Malawi Standard DHS, 2015	16462

ML	Mali Standard DHS, 2012	9582
	Mali Standard DHS, 2018	9275
MZ	Mozambique Standard AIS, 2015	5013
	Mozambique Standard DHS, 2011	10291
MM	Myanmar Standard DHS, 2015	4597
NM	Namibia Standard DHS, 2006	4858
	Namibia Standard DHS, 2013	4818
NP	Nepal Standard DHS, 2006	5457
	Nepal Standard DHS, 2011	5054
	Nepal Standard DHS, 2016	4861
NI	Niger Standard DHS, 2012	11602
NG	Nigeria Standard DHS, 2008	25446
	Nigeria Standard DHS, 2013	28596
	Nigeria Standard DHS, 2018	30713
PK	Pakistan Standard DHS, 2006	8448
	Pakistan Standard DHS, 2017	9867
PE	Peru Continuous DHS, 2009	10041
PH	Philippines Standard DHS, 2008	6382
	Philippines Standard DHS, 2017	10297
RW	Rwanda Interim DHS, 2007	5094
	Rwanda Standard DHS, 2010	8484
	Rwanda Standard DHS, 2014	7558
	Rwanda Standard DHS, 2019	2159
SN	Senegal Continuous DHS, 2012	6540
	Senegal Continuous DHS, 2014	13066
	Senegal Continuous DHS, 2014	6526
	Senegal Continuous DHS, 2015	6602
	Senegal Continuous DHS, 2016	6417
	Senegal Continuous DHS, 2017	11605
	Senegal Continuous DHS, 2018	6445
	Senegal Continuous DHS, 2019	5899

	Senegal Standard DHS, 2010-11	11633
SL	Sierra Leone Standard DHS, 2008	5043
	Sierra Leone Standard DHS, 2013	10618
	Sierra Leone Standard DHS, 2019	9063
TJ	Tajikistan Standard DHS, 2012	4838
	Tajikistan Standard DHS, 2017	6019
TZ	Tanzania Standard DHS, 2010	7496
	Tanzania Standard DHS, 2015	9713
TL	Timor-Leste Standard DHS, 2009	9294
	Timor-Leste Standard DHS, 2016	6956
TG	Togo Standard DHS, 2013	6535
UG	Uganda Standard DHS, 2006	7593
	Uganda Standard DHS, 2011	7355
	Uganda Standard DHS, 2016	14710
ZM	Zambia Standard DHS, 2007	5844

Table S2 Characteristics of the studied population.

	Overall	Control	Case
N	1281847	1221237	60610
	Mean (Standard Deviation)		
Child age (Month)	29.18 (17.30)	29.31 (17.33)	26.61 (16.40)
Life-course nighttime light (ND)	9.64 (14.10)	9.66 (14.06)	9.41 (14.91)
Maternal age (Year)	26.48 (6.32)	26.47 (6.31)	26.74 (6.40)
Interpregnancy interval (Month)	40.50 (24.59)	40.49 (24.59)	40.79 (24.59)
headage (Year)	42.32 (14.19)	42.38 (14.20)	41.20 (13.92)
breastfeed (%)			
No	46648 (3.6)	44895 (3.7)	1753 (2.9)
Yes	1130837 (88.2)	1075005 (88.0)	55832 (92.1)
Unknown	104362 (8.1)	101337 (8.3)	3025 (5.0)
Sex: Male (%)	652699 (50.9)	620523 (50.8)	32176 (53.1)
Caesarean section			
No	1124142 (87.7)	1072199 (87.8)	51943 (85.7)
Yes	134824 (10.5)	127662 (10.5)	7162 (11.8)
Unknown	22881 (1.8)	21376 (1.8)	1505 (2.5)
Place of delivery			
Home	424002 (33.1)	401917 (32.9)	22085 (36.4)
Hospital	663828 (51.8)	634431 (51.9)	29397 (48.5)
Other	4686 (0.4)	4406 (0.4)	280 (0.5)
Private	167555 (13.1)	159722 (13.1)	7833 (12.9)
Unknown	21776 (1.7)	20761 (1.7)	1015 (1.7)
Antenatal care attendance			
No	802554 (62.6)	759417 (62.2)	43137 (71.2)
Yes	114556 (8.9)	109436 (9.0)	5120 (8.4)
Unknown	364737 (28.5)	352384 (28.9)	12353 (20.4)
Singleton birth: No	29847 (2.3)	28374 (2.3)	1473 (2.4)
Nulliparous: Yes	358275 (27.9)	342456 (28.0)	15819 (26.1)

Maternal BMI			
Underweight	516957 (40.3)	490506 (40.4)	26451 (43.6)
Normal	560070 (43.7)	536438 (43.9)	23632 (39.0)
Overweight	147651 (11.5)	140455 (11.5)	7196 (11.9)
Obese	57169 (4.5)	53838 (4.4)	3331 (5.5)
Maternal employment			
No	381300 (29.7)	362035 (29.6)	19265 (31.8)
Yes	551104 (43.0)	521145 (42.7)	29959 (49.4)
Unknown	349443 (27.3)	338057 (27.7)	11386 (18.8)
Sex of household head: Male	1059537 (82.7)	1010385 (82.7)	49152 (81.1)
Wealth of household			
Poor	626220 (48.9)	596139 (48.8)	30081 (49.6)
Middle	250043 (19.5)	238377 (19.5)	11666 (19.2)
Rich	405584 (31.6)	386721 (31.7)	18863 (31.1)

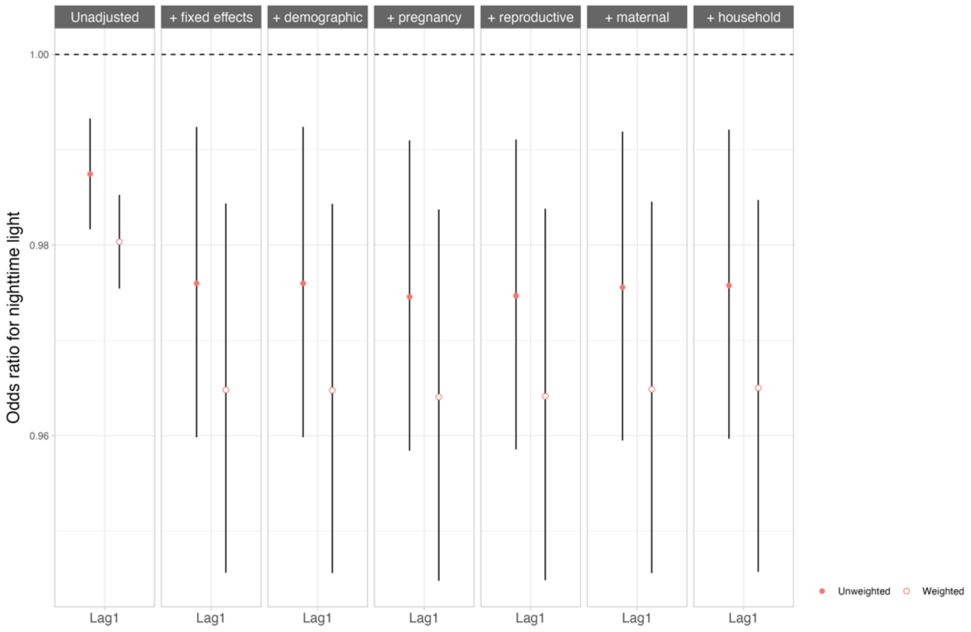

Figure S1. The average linear association between economic status and acute respiratory infection in children under five was estimated using different models.

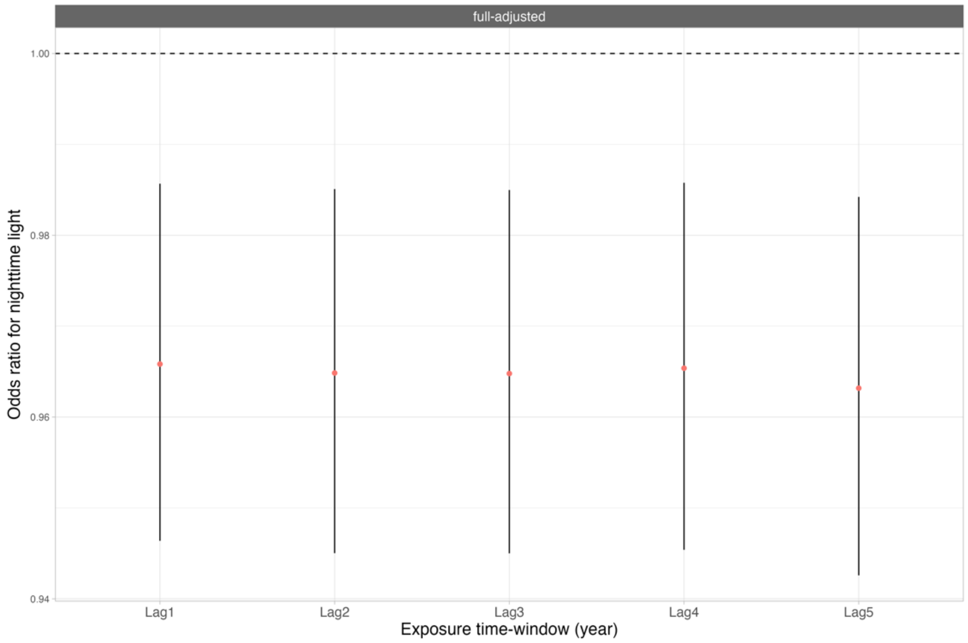

Figure S2. The full-adjusted models for the association between economic status and acute respiratory infection in children under 5 during different time-windows of exposure.

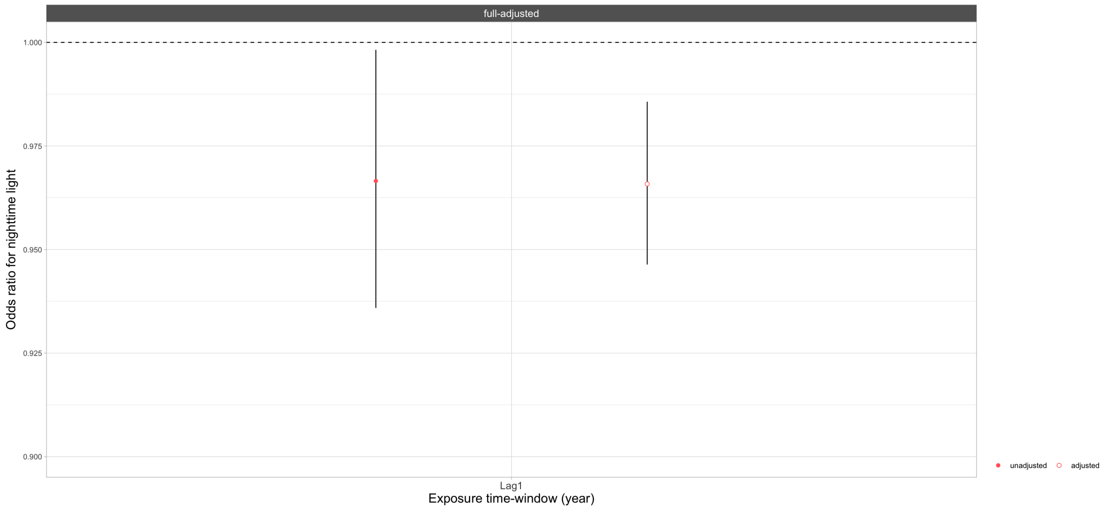

Figure S3. The association between economic status and acute respiratory infection in children under 5 was analyzed both unadjusted and after adjusting for medical treatment or advice. (The solid red circle in the figure represents the unadjusted medical treatment or advice, while the hollow red circle represents the adjusted medical treatment or advice.)

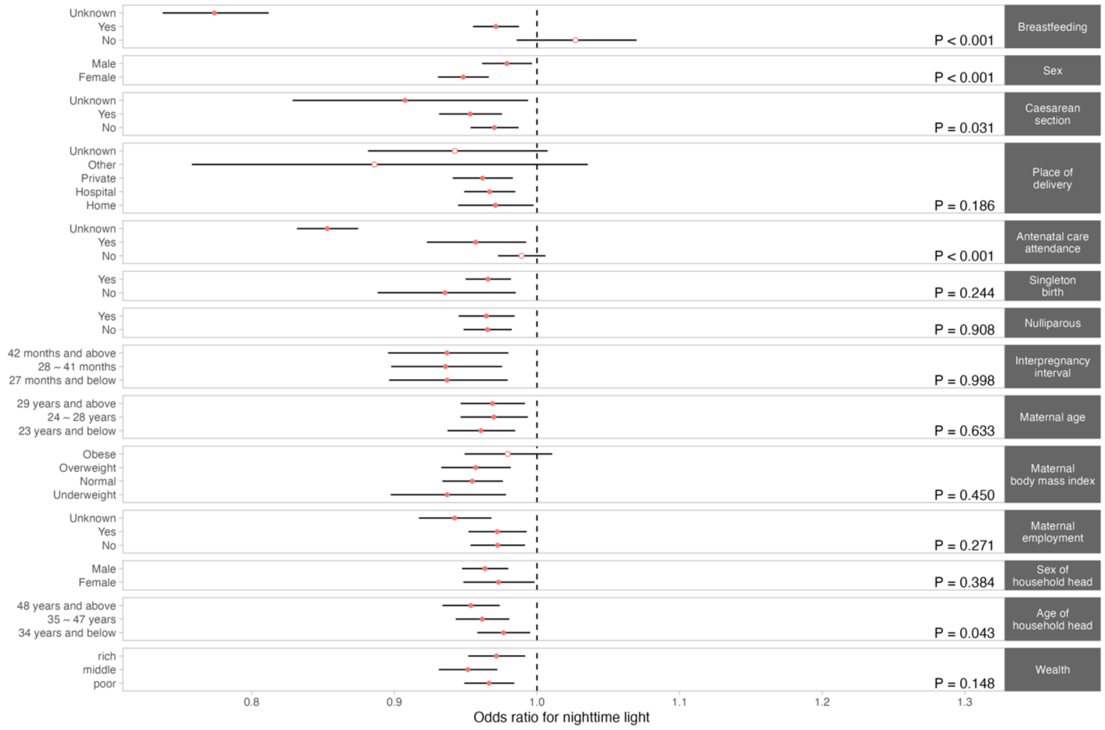

Figure S4. The association between economic status and acute respiratory infection in children under 5, estimated by different subpopulations.

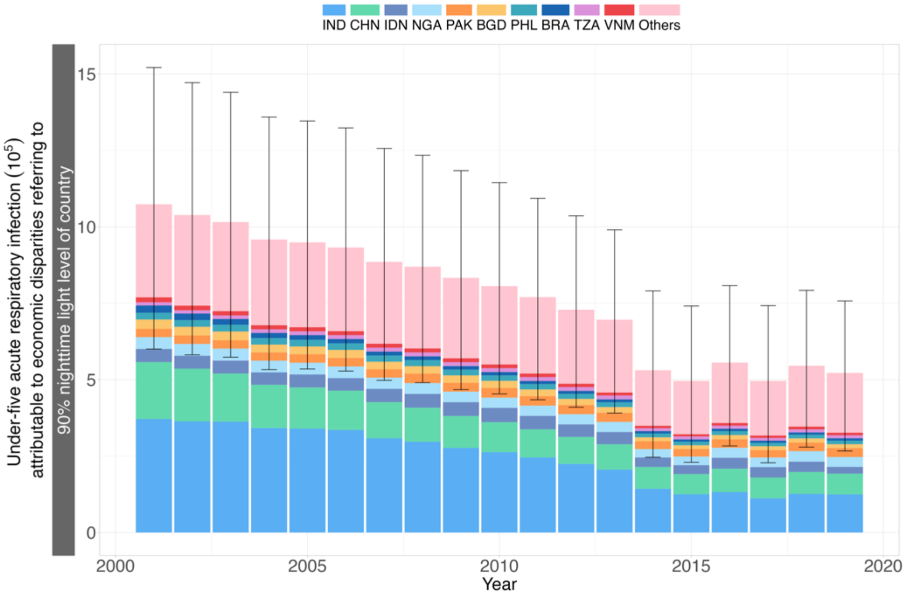

Figure S5. The top ten countries ranked by the number burden of acute respiratory infection in children under five attributed to within-country economic disparities. IND: India; CHN: China; IDN: Indonesia; NGA: Nigeria; PAK: Pakistan; BGD: Bangladesh; PHL: Philippines; BRA: Brazil; TZA: Tanzania; VNM: Vietnam.

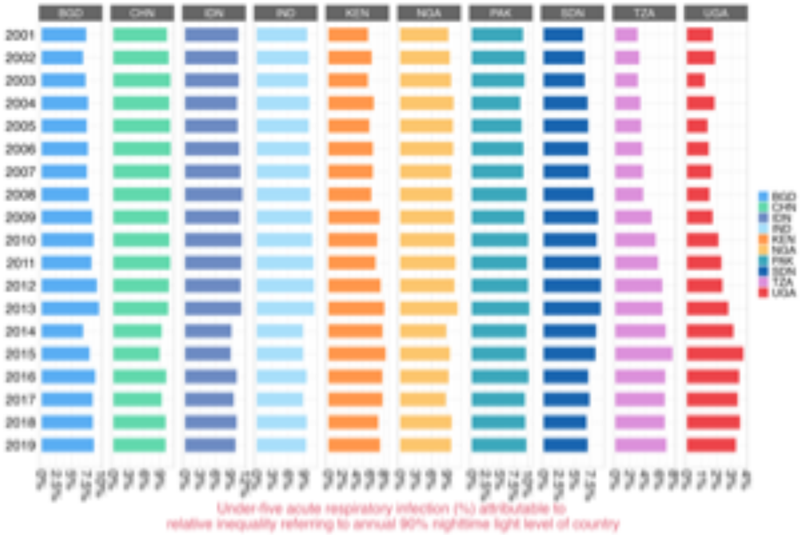

Figure S6. The burden of acute respiratory infection in children under five attributed to within-country relative economic inequality in ten countries. BGD: Bangladesh; CHN: China; IDN: Indonesia; IND: India; KEN: Ethiopia; NGA: Nigeria; PAK: Pakistan; SDN: Sudan; TZA: Tanzania; UGA: Uganda.

(a)

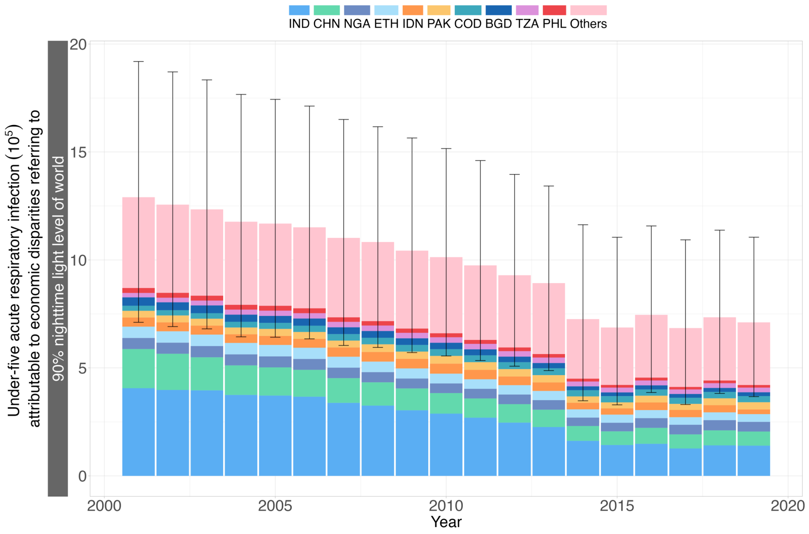

(b)

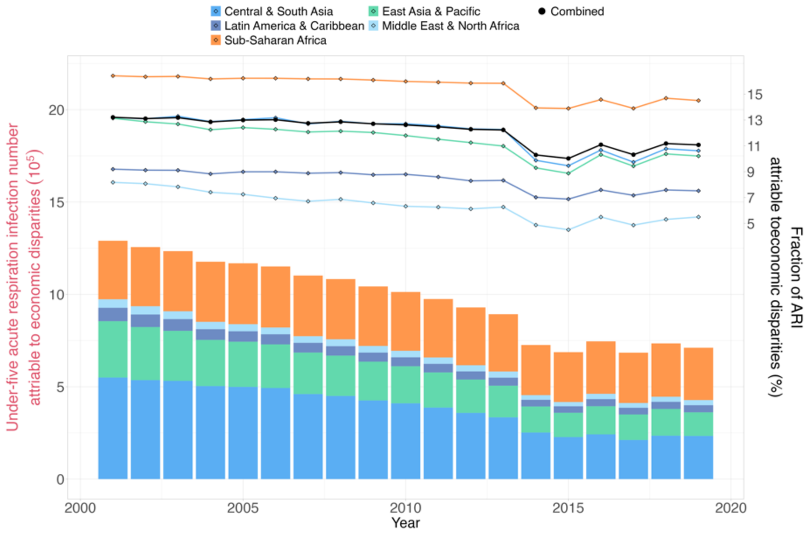

Figure S7. (a) The top ten countries ranked by the number burden of acute respiratory infection in children under five attributed to within-world economic disparities. IND: India; CHN: China; NGA: Nigeria; ETH: Ethiopia; IDN: Indonesia; PAK: Pakistan; COD: Congo; BGD: Bangladesh; TZA: Tanzania; PHL: Philippines. (b) The temporal trends of acute respiratory infection burden in children under five are attributable to within-world economic disparities, segmented by geographic location. The left y-axis represents the attributable numbers (colored bars), and the right y-axis represents the attributable fractions (circles and lines).

(a)

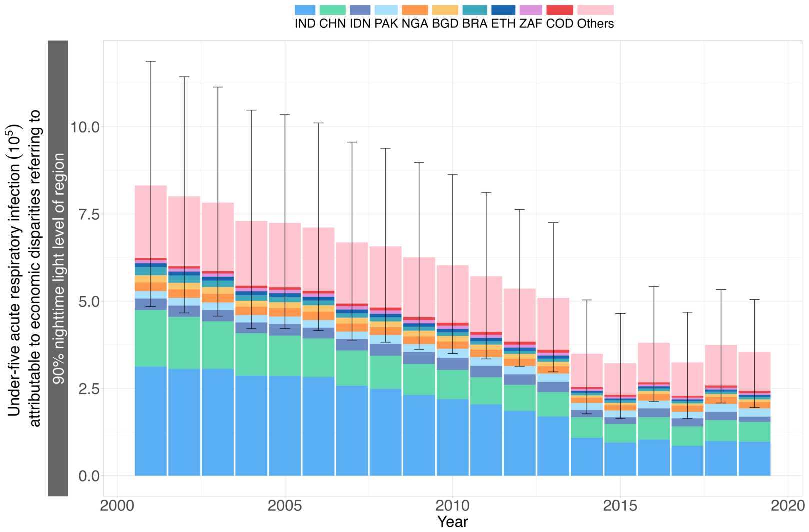

(b)

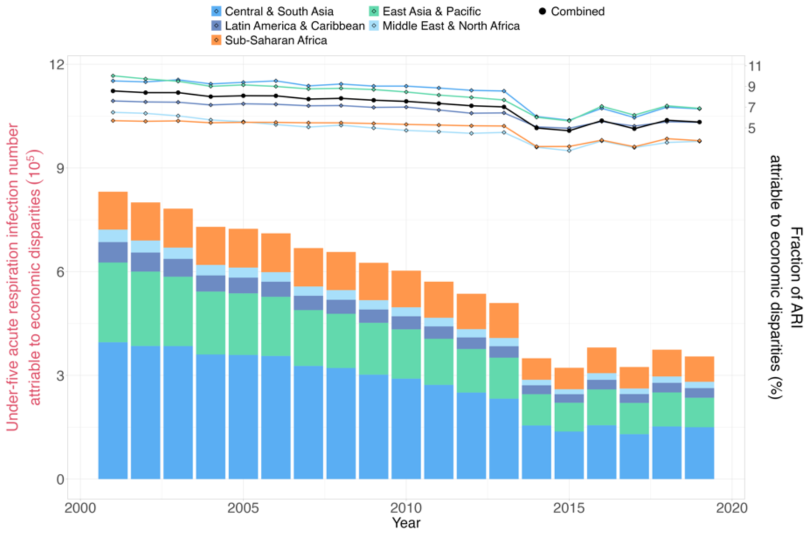

Figure S8. (a) The top ten countries ranked by the number burden of acute respiratory infection in children under five attributed to within-region economic disparities. IND: India; CHN: China; IDN: Indonesia; PAK: Pakistan; NGA: Nigeria; BGD: Bangladesh; BRA: Brazil; ETH: Ethiopia; ZAF: South Africa; COD: Congo. (b) The temporal trends of acute respiratory infection burden in children under five are attributable to within-region economic disparities, segmented by geographic location. The left y-axis represents the attributable numbers (colored bars), and the right y-axis represents the attributable fractions (circles and lines).

(a)

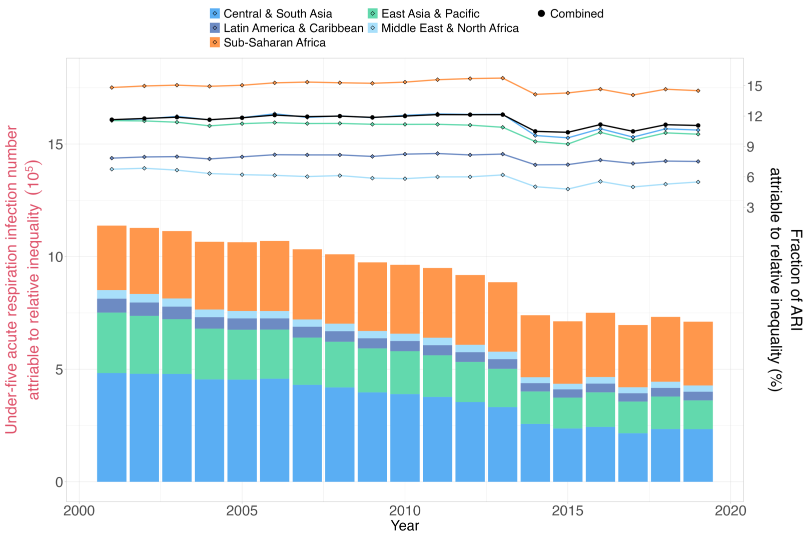

(b)

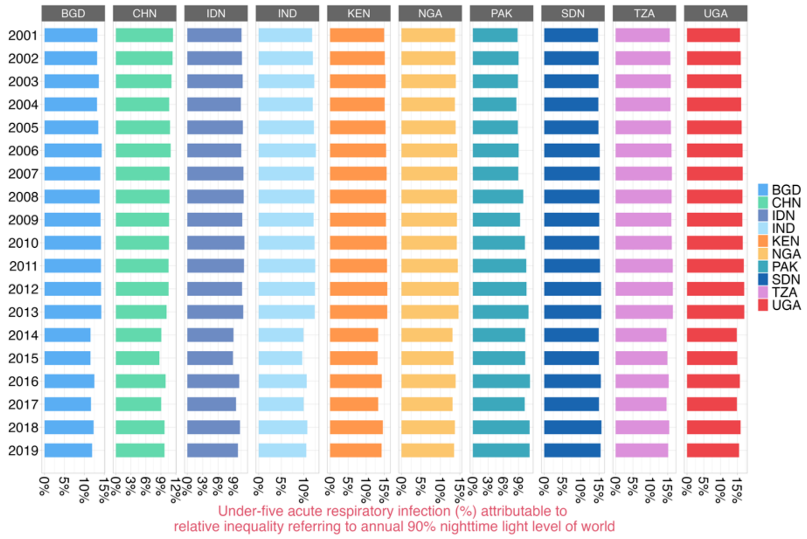

Figure S9. (a) The temporal trends of acute respiratory infection burden in children under five, attributed to within-world relative economic inequality, are segmented by geographic location. The left y-axis represents the attributable numbers (colored bars), and the right y-axis represents the attributable fractions (circles and lines). (b) The burden of acute respiratory infection in children under five attributed to within-world relative economic inequality in ten countries. BGD: Bangladesh; CHN: China; IDN: Indonesia; IND: India; KEN: Ethiopia; NGA: Nigeria; PAK: Pakistan; SDN: Sudan; TZA: Tanzania; UGA: Uganda.

(a)

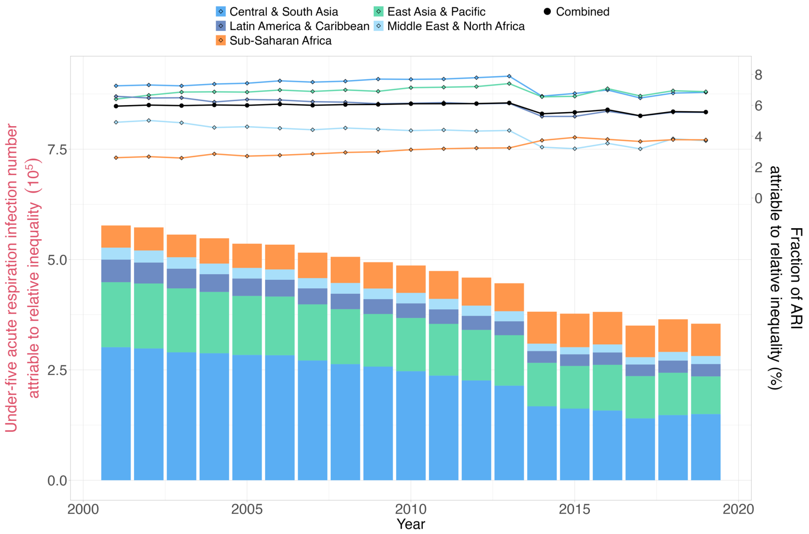

(b)

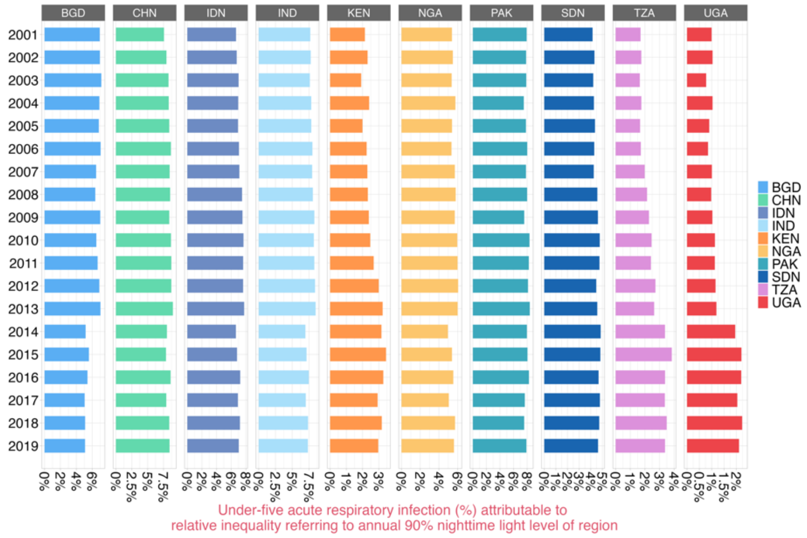

Figure S10. (a) The temporal trends of acute respiratory infection burden in children under five, attributed to within-region relative economic inequality, are segmented by geographic location. The left y-axis represents the attributable numbers (colored bars), and the right y-axis represents the attributable fractions (circles and lines). (b) The burden of acute respiratory infection in children under five attributed to within-region relative economic inequality in ten countries. BGD: Bangladesh; CHN: China; IDN: Indonesia; IND: India; KEN: Ethiopia; NGA: Nigeria; PAK:

Pakistan; SDN: Sudan; TZA: Tanzania; UGA: Uganda.

(a)

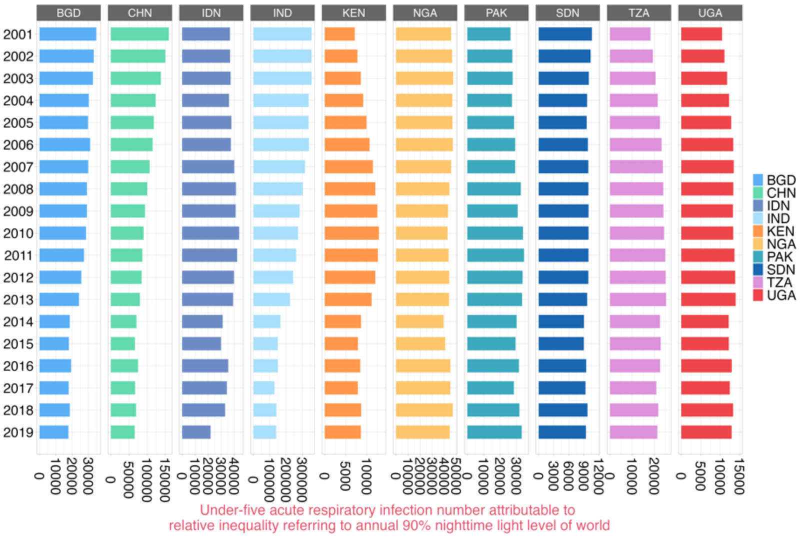
BGD CHN IDN IND KEN NGA PAK SDN TZA UGA
2001 2002 2003 2004 2005 2006 2007 2008 2009 2010 2011 2012 2013 2014 2015 2016 2017 2018 2019
BGD
CHN
IDN
IND
KEN
NGA
PAK
SDN
TZA
UGA
Under-five acute respiratory infection number attributable to
relative inequality referring to annual 90% nighttime light level of world

(b)

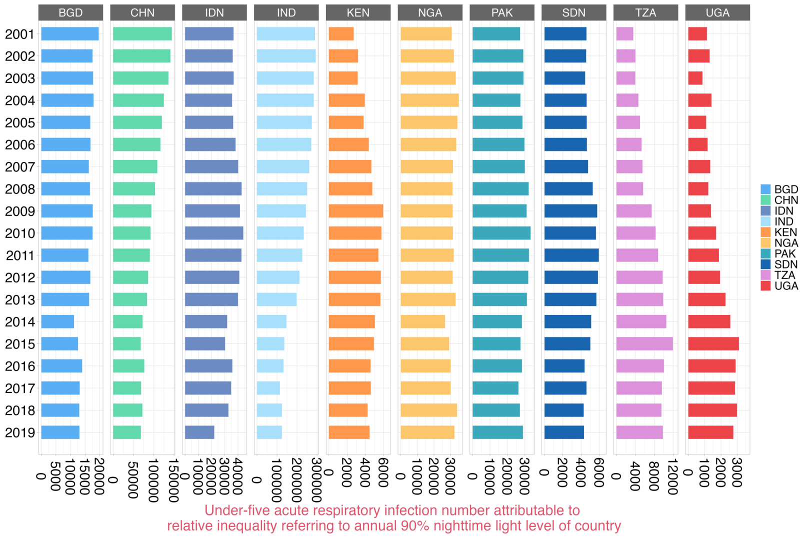
BGD CHN IDN IND KEN NGA PAK SDN TZA UGA
2001 2002 2003 2004 2005 2006 2007 2008 2009 2010 2011 2012 2013 2014 2015 2016 2017 2018 2019
BGD
CHN
IDN
IND
KEN
NGA
PAK
SDN
TZA
UGA
Under-five acute respiratory infection number attributable to
relative inequality referring to annual 90% nighttime light level of country

(c)

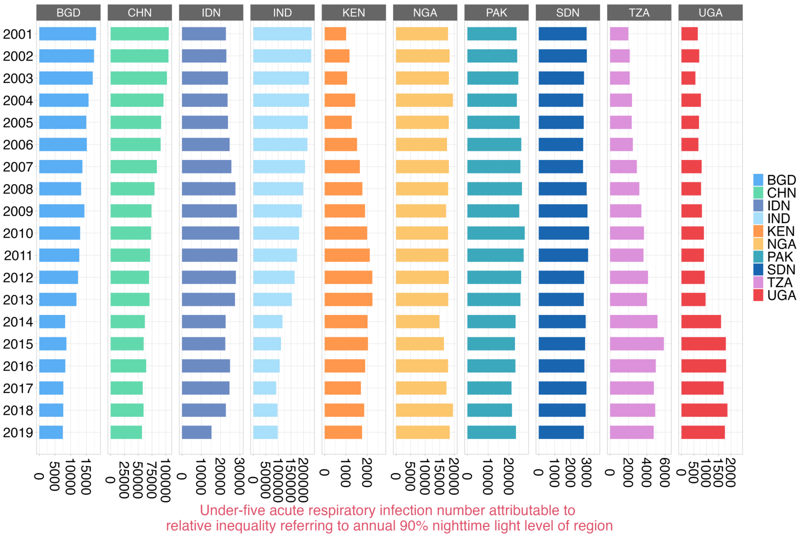
BGD CHN IDN IND KEN NGA PAK SDN TZA UGA
2001 2002 2003 2004 2005 2006 2007 2008 2009 2010 2011 2012 2013 2014 2015 2016 2017 2018 2019
BGD
CHN
IDN
IND
KEN
NGA
PAK
SDN
TZA
UGA
Under-five acute respiratory infection number attributable to
relative inequality referring to annual 90% nighttime light level of region

Figure S11. (a) The number burden of acute respiratory infection in children under five attributed to within-world relative inequality, for ten countries. BGD: Bangladesh; BRA: Brazil; CHN: China; EGY: Egypt; IND: India; IDN: Indonesia; KEN: Ethiopia; NGA: Nigeria; PAK: Pakistan; TZA: Tanzania. (b) The number burden of acute respiratory infection in children under five attributed to within-country relative inequality, for ten countries. BGD: Bangladesh; BRA: Brazil; CHN: China; EGY: Egypt; IND: India; IDN: Indonesia; KEN: Ethiopia; NGA: Nigeria; PAK: Pakistan; TZA: Tanzania. (c) The number burden of acute respiratory infection in children under five attributed to within-region relative inequality, for ten countries. BGD: Bangladesh; BRA: Brazil; CHN: China; EGY: Egypt; IND: India; IDN: Indonesia; KEN: Ethiopia; NGA: Nigeria; PAK: Pakistan; TZA: Tanzania.

Text 1: Covariates

Based on previous impact studies of ARI in children under the age of 5, we have considered the following factors:

i) Demographic: Sex of child (female or male); Breastfeeding status (still breastfeeding or not)

ii) Pregnancy: Caesarean section (yes ,or no, or unknown); Place of delivery (home, or hospital, or private, or other, or unknown); Antenatal care attendance (yes ,or no, or unknown)

iii) Reproductive: Singleton birth (yes or no); Nulliparous (yes or no) ; Interpregnancy interval (re-coded into three categories: “27 months and below”, “28 ~ 41 months”, “42 months and above”)

iv) Maternal: Maternal age (re-coded into three categories: “23 years and below”, “24 ~ 28 years”, “29 years and above”); Maternal body mass index (re-coded into four categories: “Underweight”, “Normal”, “Overweight” , “Obese”); Maternal employment (yes, or no, or unknown)

v) Household: Sex of household head (female or male); Age of household head (re-coded into three categories: “34 years and below”, “35 ~ 47 years”, “48 years and above”); Household wealth status (re-coded into three categories: “poor”, “middle”, “rich”).

Text 2: Statistical analyses

Firstly, in order to comprehensively understand the association between economic status and acute respiratory infection, a varying-coefficient regression model was applied to obtain the nonlinear exposure response relationship using the DHS data from 53 countries. Secondly, to quantify the burden of ARI of children under 5 years attributable to relative economic inequality in 133 low-and middle-income countries, we utilized the nonlinear exposure response relationship derived from first step to conduct risk assessment. When performing point estimates, the use of different reference values did not change the curvature of the exposure response relationship, but only caused a vertical shift in the graph. Therefore, in this study, it was only necessary to conduct the appropriate numerical conversions for calculating attributable fractions using different reference values.

Multicenter analysis, including this study, usually involve two stages: first, the sampling strata are selected and defined as the centers; and second, within each stratum, samples are obtained as representative for the local population[1-3]. Therefore, the multicenter features of the sampling design should be considered in the association model, resulting in a fixed-effects model. The parameterization of this model is presented below:

$$Logit(y_{i,j}) = x_{i,j}\beta + z_{i,j}\gamma_i + \alpha_i + e_{i,j} \quad (1)$$

In this equation, i is the index for strata, j denotes the index for subjects, $x_{i,j}$ stands for the level of nighttime light, $z_{i,j}$ represents the adjusted covariates, β and γ_i are the corresponding regression coefficients, α_i denotes the strata-specific intercept in strata, and $e_{i,j}$ represents residual term.

In equation (1), this study adjusted for multiple covariate types, including characteristics related to demographics, pregnancy, reproductive, maternal, and household. Based on the fundamental concept of the multi-center fixed-effects model, we further rewrite Equation (1) as follows:

$$Logit(y_{i,j}) = (x_{i,j} - x_i)\beta + z_{i,j}\gamma_i + \alpha_i + x_i\beta + e_{i,j} = r_{i,j}\beta + z_{i,j}\gamma_i + \alpha_i^* + e_{i,j}$$
$$x_i = \sum_j x_{i,j}/n_i \, , r_{i,j} = x_{i,j} - x_i, \alpha_i^* = \alpha_i + x_i\beta \qquad (2)$$

In this equation , x_i stands for the strata-level mean of nighttime light, $r_{i,j}$ is derived from the mean, α_i^* represents an alternative fixed effect for each stratum. As shown in the formula, equation (1) and equation (2) are equivalent. The new equation utilizes $r_{i,j}$ to represent the exposure variation within strata. In the context of this large-scale multicenter study, the independent variable $r_{i,j}$ stands for the marginal change when contrasted with the between-strata variation.

In order to acquire a non-linear exposure-response relationship, this study replaced the exposure effect $x_{i,j}\beta$ in equation (1) with a functional term, $f(x_{i,j})$, resulting in a new model:

$$Logit(y_{i,j}) = f(x_{i,j}) + z_{i,j}\gamma + \alpha_i + e_{i,j} \qquad (3)$$

As the formula indicates, there is a potential issue of multicollinearity between $f(x_{i,j})$ and α_i. To tackle this challenge, this study utilized the marginal variation of nighttime light, as it can be approximated by a first-order Taylor polynomial expansion of f(x). The modified equation (3) is

presented below:

$$Logit(y_{i,j}) = f(x_i + r_{i,j}) + z_{i,j}\gamma + \alpha_i + e_{i,j} \ = f'(x_i)r_{i,j} \ + f(x_i) + z_{i,j}\gamma + \alpha_i + e_{i,j}$$

$$= f^{'(x_i)r_{i,j}} + z_{i,j}\gamma + \alpha_i^{**} + e_{i,j} \ , \ \alpha_i^{**} \ = \alpha_i + f(x_i) \ (4)$$

where α_i^{**} represents a new fixed effect and $f'(x_i)r_{i,j}$ denotes the first derivative of the non-linear function derived from the average within each stratum. Hence, we re-evaluated the exposure-response relationship based on equation (4). Next, given a predetermined reference, we applied an integration to obtain the effect for an increase from reference to a given level of nighttime light (nonlinear ERF). We predetermined a reference and applied the integration of $f'(x_i)$ to re-examine the model:

$$f(x) = \int_{\text{reference}}^{x} f'(x)\, dx \qquad (5)$$

[1] Basagaña X et al. Analysis of multicentre epidemiological studies: contrasting fixed or random effects modelling and meta-analysis. *Int J Epidemiol.* 2018;47(4):1343-1354.

[2] Xue T et al. Estimation of stillbirths attributable to ambient fine particles in 137 countries. *Nat Commun.* 2022;13(1):6950.

[3] Xue, T et al. Estimating the exposure-response function between long-term ozone exposure and under-5 mortality in 55 low-income and middle-income countries: a retrospective, multicentre, epidemiological study. *The Lancet Planetary health*.2023;7(9): e736–e746.
